# Causality-Sensitive Scheduling to Reduce Latency in Vehicle-to-Vehicle Interactions

**DOI:** 10.3390/s24227142

**Published:** 2024-11-06

**Authors:** Hojeong Lee, Seungmo Kang, Hyogon Kim

**Affiliations:** Department of Computer Science and Engineering, Korea University, Anam-Dong, Sungbuk-Gu, Seoul 02841, Republic of Korea; hojeong0507@korea.ac.kr (H.L.); seungmo@korea.ac.kr (S.K.)

**Keywords:** vehicle-to-vehicle (V2V), cellular V2X, sidelink, interactive applications, resource allocation, delay spikes elimination

## Abstract

This paper shows through real-life measurement that bi-directional vehicle-to-vehicle (V2V) communication latency can be dominated by sidelink scheduling delay when causality is not taken into account. Moreover, the large delay persists for a few seconds at a time once it occurs. In applications like maneuver coordination between autonomous vehicles or in platoon, such delay can be highly detrimental to safety and efficiency. We investigate the source of the problem and propose a solution that factors in causality in interactive communication. Specifically, we develop a constraint under which the resource positions are automatically aligned between the communicating vehicles, and the delay spikes are provably eliminated. Through the measurements on commercial V2X devices, we confirm that enforcing the constraint can remove latency spikes so that 5G sidelink can be more easily applied to time-sensitive interactions between vehicles.

## 1. Introduction

As the number of connected vehicles increases, vehicular networking is rapidly becoming a reality. Vehicular networking enables a wide range of applications, including safety, traffic efficiency, and infotainment [1]. In the safety category, applications like collision avoidance, road hazard warnings, and lane-change assistance exchange messages with the highest priority among vehicular networking applications. Next, applications like safe distance maintenance, traffic light schedule information, route guidance, and parking information are supported. Finally, infotainment includes access to local information, maps, and internet usage, with messages exchanged at the lowest priority level.

Vehicular networks use wireless technology for connectivity due to the high mobility of vehicles. Depending on applications, the traffic on the network can be delivered in multiple hops or a single hop. Information dissemination type of applications such as precautions or traffic information can benefit from multi-hop communication as the region of interest can exceed the usual single-hop communication range. Vehicular ad-hoc network (VANET) [2] is a well-studied topic in the multi-hop vehicular network environments where issues like link disconnections and quality degradation, heterogeneity in network types, fluctuating traffic density, and routing efficiency must be resolved. For example, when the network disconnections render the end-to-end path difficult, a delay-tolerant network (DTN) can be employed to achieve the data delivery [3], which is at one extreme of the vehicular communication delay spectrum.

For driving safety in short regions of interest, such as between neighboring vehicles, however, single-hop communication is the predominant choice. In particular, future autonomous vehicles will directly communicate with each other to convey driving intentions and negotiate the best maneuvers. This type of communication is at the opposite end of the delay spectrum. In fact, single-hop communication is defined in most vehicular communication standards such as IEEE WAVE [4,5] or cellular V2X [6,7] as the major enabling technology. Unsurprisingly, the European New Car Assessment Program (EuroNCAP) 2025 Roadmap [8] shows a long-term commitment to vehicular communication. It brought a real advancement for intelligent transportation systems (ITS), enabling vehicles to communicate with each other (V2V), infrastructure (V2I), and even pedestrians (V2P) to enhance road safety, traffic efficiency, and user convenience. Their relevance has grown as connected vehicles are envisioned as an integral part of smart cities [9], impacting both public safety and infrastructure management.

Among vehicular communication, V2V communication is anticipated to be the most critical technology for a range of future applications, including autonomous driving, autonomous vehicular platooning, and connected assisted driving, aiming to enhance driving safety and traffic efficiency. For instance, it can help mitigate the issues with critical line-of-sight (LoS) sensor malfunctions [10] or dangerous decisions made by faulty autonomous driving logic [11]. In terms of traffic efficiency, it could address the current situation that AVs are programmed to move more conservatively compared to human drivers, which can increase travel time [12]. In autonomous vehicular platooning, V2V communication not only enhances traffic flow but also significantly lowers the risk of rear-end collisions, as the vehicles can react instantaneously to the actions of the lead vehicle [13,14,15]. Obviously, the biggest issue in these applications is the communication latency. Due to the characteristics of incidental encounters, high speeds, and the resulting risk of accidents, V2V communication must achieve minimal interaction delay, regardless of the circumstances under which communication occurs. In the context of connected assisted driving, vehicle-to-everything (V2X) communication extends beyond V2V to include vehicle-to-infrastructure (V2I) and vehicle-to-pedestrian (V2P) interactions. It allows for real-time updates on road hazards or pedestrian movements, therefore enhancing overall driving safety.

Inter-vehicle communication has traditionally relied on unilateral broadcasting as its base mode, supplemented by event-based messaging. This approach ensures a constant stream of information while allowing for specific, timely updates when particular events occur. In the “Day 1” use case scenario, it is the ego vehicle kinematics information [16] that is broadcast to raise the awareness of the vehicle among its closely located neighbors. In “Day 2”, it is the road users’ information obtained by the ego vehicle’s sensors [17] that is broadcast to be used by neighbors to extend their perception range. In “Day 3”, which is the use case scenario for AVs, it is the ego vehicle’s driving intention in terms of its current (“planned”) trajectory and intended (“desired”) trajectory [18] that is broadcast. The corresponding messages to these use case scenarios are Cooperative Awareness Message (CAM) [16], Collective Perception Message (CPM) [17], and Maneuver Coordination Message (MCM) [18], respectively.

Similar to Day 1 and Day 2 messages, MCM packets are unilaterally and periodically broadcast at a rate between 1 and 10 Hz. Unfortunately, these periodic broadcast messaging in cellular V2X environments raises a serious communication delay on Day 3. This is because the application characteristics are different from Day 1 and Day 2. On Day 3, autonomous vehicles can more explicitly interact in a request-response manner through MCM. Specifically, vehicles request trajectory changes through periodic MCMs, and the vehicles that need to respond to these requests also transmit the MCMs on their own periodic schedule. Similarly, in platooning, Platoon Control Messages (PCMs) are exchanged periodically between the platoon member and leader at a higher frequency (e.g., 20 Hz) [19]. Specifically, PCMs are unicast [20] or possibly broadcast [19], first by the platoon leader and subsequently by the platoon member. Then, in the wireless resource reservation method for periodic transmission in the cellular V2X environments, the resources of both interacting parties are allocated independently without considering the interaction. Consequently, the interaction delay can be significantly longer than the possible minimum time.

However, it is not without reason that the periodic transmission was chosen as the base mode for Day 3. Compared with explicit communication, periodic message exchange also has advantages. First, The continuous message exchange enables early detection of the need for maneuver coordination [19]. Second, the periodic (hence repetitive) message exchange is more robust against communication errors, such as the Two Generals’ Problem [21]. It is notable that although event-based message transmission has been added recently to enhance the system [22], the base mode of communication remains periodic. Therefore, in this paper, we aim to find a way to minimize the delay in vehicle interactions while maintaining the advantages of periodic messaging.

We proceed in the following way. First, we confirm through actual experimentation with commercial V2X devices that the exchange of the current unilateral broadcast messaging does not accommodate the bilateral nature of maneuver coordination well. The reason for this is that the causality in the coordination process is not reflected in the communication schedule and leads to unnecessary delays in the cellular V2X communication environment. Such delay appears unpredictably, and the excessive delay is maintained for a few seconds once the delay spike breaks out. Also, the delay fluctuates severely due to the independent resource reselection procedures on both vehicles. Then, we investigate the mechanism behind the delay spikes and propose a solution to remove them. By considering the causality of the application on the message exchange schedule in terms of the communication resource allocation, we find that such undesirable delay spikes can be deterministically eliminated. As a consequence, the mean V2V transaction delay is reduced from over 90 ms to less than 20 ms (approximately 80% reduction), and the standard deviation from nearly 40 ms to less than 5 ms (approximately 85% reduction). This could be achieved by only slightly reducing the packet delay budget (PDB) below the resource reservation period, which is then leveraged to trigger a resource reselection before the delay spike occurs. This paper discusses this solution approach as well as other arrangements to be made in addition to the PDB adjustment.

To summarize, the contributions of this paper are as follows:

- Demonstrates through real-life V2X devices that vehicle-to-vehicle interaction can suffer unpredictable delay fluctuations unless causality is considered in resource scheduling, which may threaten time-critical cooperation.
- Develops a systematic and provable method to avoid such delay fluctuations.
- Demonstrates through simulation and actual measurement on commercial V2X devices that the proposed method does eliminate excessive delay fluctuations.
- Demonstrates that interactive vehicular applications can achieve low delay also through periodic messaging, obviating the need to resort to event-driven messaging for delay reasons. To the best of our knowledge, this work is the first to show it.

The rest of the paper is organized as follows. Section 2 discusses the related work on the delay issues in interactive communication between vehicles. It also provides background on resource allocation in the cellular V2X environments. Section 4 describes the delay spikes issue for vehicles interacting through 3GPP sidelink communication. It also presents the real-life measurement data that demonstrates the delay spikes. Section 5 presents a solution based on the PDB-triggered resource reselection along with the reduced selection window size. It derives the range of PDB values to be used to trigger such reselection. Section 5.2 demonstrates through measurement and simulation that the proposed solution is effective in resolving the delay spikes. Finally, Section 6 concludes the paper.

## 2. Related Work

There is a rich literature on vehicular communication. Vehicular ad-hoc network (VANET) is an area where vehicles become nodes in an information flow network [23]. Due to the possibility of multi-hop data transfer, issues like routing [24], security [25], multi-hop transfer in the face of intermittent connection [3], quality of service (QoS) [26], and scalability [27] become issues. Although VANET covers both multi-hop and single-hop communication, most current and commercial developments, as well as standards, focus on single-hop communication that is geared towards driving safety. Our work also addresses single-hop communication. In particular, we investigate the latency aspect of real-time vehicle-to-vehicle interactions, such as those expected between autonomous vehicles [18].

When computing on vehicles is necessary to support real-time vehicular applications, where vehicles offload intensive computation tasks to the nearby edge computing server for processing [28]. In the process, the communication resource allocation should also be considered to optimize the offloading decision. Our work departs from these joint computation-communication optimization works in two points. First, the computation is minimal in our setting and the communication is directly done through the sidelink, thus does not involve the edge at all. In fact, the sidelink communication must work when there is no infrastructure support (edge or base station). Second, the resources that we deal with are solely communication resources.

3GPP sidelink communication latency for vehicles cooperating through periodic messages can be critical in V2X-supported applications that require real-time interaction, e.g., autonomous driving, platooning [29], V2X-assisted autonomous driving [30]. In autonomous driving, each vehicle primarily relies on information it collects through its own onboard sensors. To overcome the limitations of the single-agent nature of autonomous driving, however, a V2X-enabled collaborative autonomous driving framework can be employed to improve safety. He et al. [30] implemented a prototype where vehicles broadcast their information through Basic Safety Message (BSM) [31] to which RSU responds with guiding information also through BSM. On both sides, BSMs are transmitted every 100 ms.

In the European standard under development, vehicles coordinate their trajectories through an implicit protocol based on periodic MCM exchanges, in particular in the base concept [32]. Correa et al. [33] showed that maneuver coordination indeed has the potential to increase the speed of vehicles that perform coordination without degrading the overall traffic flow. Implicit coordination through periodic messaging has pros and cons compared to explicit, event-triggered messaging adopted in the U.S. [34]. The latter is bandwidth-efficient, while the former is more robust to message delivery problems on the access layer. Specifically, periodically sending the reference trajectory helps to increase the awareness of own plans to other vehicles and to find out if cooperation with other vehicles is necessary and with whom [32].

In platooning application, CAM or PCM are envisioned to be used between the platoon leader and the platoon member(s). The leader periodically transmits the CAM or PCM, which is subsequently matched by the transmissions from the members. According to Molina-Masegosa et al. [35], the traffic pattern of a platooning application entails periodic exchange of packets whose delivery requirements are the end-to-end latency in the order of 10–25 ms. Due to the low-latency requirement, Nardini et al. [36] proposed to use dynamic scheduling, which is controlled by a base station if it is present. Although limited to the platooning application, another approach to reduce communication delay inside the platoon is to let the platoon leader, delegated by a base station, take charge of the scheduling for the whole platoon [37]. These works testify to the difficulty of reducing sidelink communication delays among vehicles, especially in time-sensitive applications.

In this paper, we consider the distributed resource scheduling in cellular V2X environments that automatically adjusts the resource positions so that the interactive communications, as discussed above, suffer minimal delay on the sidelink. Unlike existing works, we particularly focus on the impact of resource reselection procedures on interaction delays. By leveraging on the standard-compliant PDB-triggered reselection, we demonstrate that we can deterministically control the communication latency in inter-vehicle interactions even when we use periodic messaging and distributed scheduling.

## 3. Background: Periodic Resource Allocation in Cellular V2X Communication

In the cellular V2X communication using periodic broadcast, the Sensing-Based Semi-Persistent Scheduling (SPS) algorithm [6] is executed at each vehicle for distributed resource allocation. As base stations may not be present in some locations, the distributed scheduling is the base mode and is called the sidelink Mode 2 in 5G New Radio (NR). When a vehicle needs to select a resource at time slot *n*, the so-called selection window is hypothesized, which spans the time range of $\left[n+T_{1},n+T_{2}\right]$. $T_{1}$ is the processing time required for the vehicle to compute the resource location based on the observation of the past 1100 ms (for periodic transmission) or 100 ms (for aperiodic transmission). $T_{2}$ value must be within the range $T_{2min}\leq T_{2}\leq PDB$, with its value left to User Equipment (UE; OBU or RSU) implementation. The packet delay budget (PDB) is set by the application and given to the scheduling algorithm as a parameter. Once the resource is selected from the selection window, SPS allows the vehicle to use it multiple times at subframes $t_{\mathrm{TX}}+\left(k-1\right)\cdot RRP\;\;\left(1\leq k\leq RC\right)$. Here, $t_{\mathrm{TX}}$ is the first subframe when the vehicle starts sending the packet with the reserved resource, RRP is the vehicle’s Resource Reservation Period (RRP), and RC is the Resource Reselection Counter. RRP can be set to 1 to 99 ms or multiples of 100 ms up to a maximum value of 1000 ms. The RC values are set to a uniform random number. For instance, RC∼Uniform[5,15] at RRP ≥ 100 ms or $Uniform\left[5\times \frac{100}{max(20,RRP)},15\times \frac{100}{max(20,RRP)}\right]$ otherwise [7]. When the RC decrements to zero, the vehicle reselects a new resource with probability $1-P_{keep}$ or continues to use the previous one with $P_{keep}\leq 0.8$. This process prevents most resource collisions by multiple vehicles.

## 4. Problem Description

In this section, we describe the problem that is addressed by the proposed scheme. We first show how the problem can materialize through timing relations analysis, then provide the evidence by measurement experiments on real-life devices.

### 4.1. Round-Trip Latency in Interactive Communication

When a vehicle makes a request and other vehicle(s) respond to it, the interaction exhibits causality. In considering causal interaction in the cellular V2X environments, we should consider two problems: scheduling delay and causal relation that affects interaction delay.

**Figure 1:**
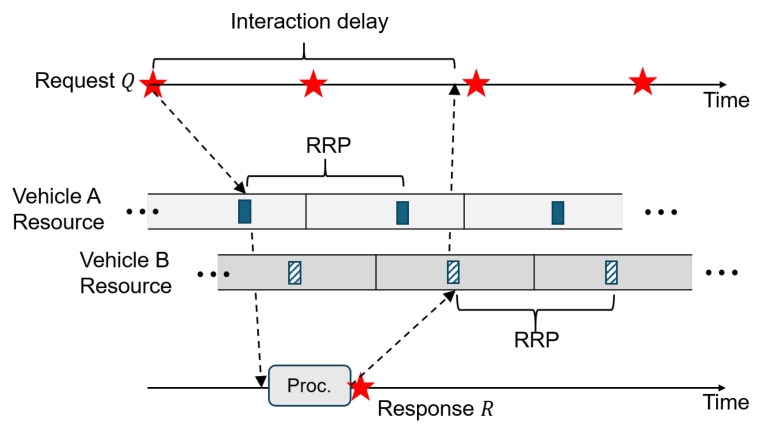
Effect of resource selection window size on transaction delay, where stars are application messages (frequency axis in the resource plane omitted for brevity).

The first problem is related to the communication delay that can be doubled compared to unilateral broadcast applications. Therefore, the delay must be more tightly controlled. For example, assume the resource is reserved for message transmissions that occur every 100 ms (i.e., RRP = 100 ms) on each side of the interacting vehicles. Then, as Figure 1 illustrates, the requesting vehicle could experience a delay close to or even exceeding 200 ms until it receives the response in the worst case. Specifically, the interaction delay is

(1) $$RTT\leq 2T_{2}+T_{proc}+2T_{t+r},$$

where $T_{t+r}$ is the transmission plus reception delay (e.g., from transmission at Vehicle A to reception at Vehicle B or vice versa) that is assumed order(s) of magnitude smaller than $T_{2}$. $T_{proc}$ is the message processing delay on the application layer required to process information from the request to produce a response. Longer RTT will be incurred if Vehicles A and B select resources located more towards the end of their selection window, i.e., $T_{2}$. In fact, we will demonstrate through measurements on commercial V2X devices that this phenomenon is frequently observable. If the RTT exceeds the message generation interval of 100 ms, Vehicle A will not receive a response from Vehicle B before its next request transmission. This will make Vehicle A send the next request without hearing Vehicle B’s response to the outstanding request. However, it would be more natural and more desirable for real-time applications that the response arrives before the next request transmission, which may depend on the contents of the most recent response. Obviously, a remedy to this phenomenon would be to reduce the *size* of the selection window, namely $T_{2}$ in Equation (1). In that case, one would argue that the RTT will be accordingly reduced because the time gap between the message generation on the higher layer and its transmission on the access layer will become much smaller.

Unfortunately, reducing $T_{2}$ in Equation (1) only partially solves the problem. This is because of an aspect not captured by the inequality. It is the delay pathology caused by the *order* of the resource topology that violates the causality of interaction. Since each vehicle independently reserves its sidelink resources without considering the relative timing of these resources in potential interactions, there is a lack of resource synchronization between interacting vehicles. Depending on the relative timing, therefore, the communication delay could become high. In essence, a delayed surge can happen when the causality of interaction on the application layer is not reflected on the resource allocation (i.e., MAC) layer. To understand this aspect, we need to classify the resource topology between communicating vehicles *A* and *B*: *forward order* and *reverse order*. Let us define the resource that first appears on A after the request $Q_{n}$ be $V_{A}^{n}$ and the resource on B be $V_{B}^{n}$. For convenience, we use $Q_{n}$ as our time reference that any event on either requestor and replier side after $Q_{n}$ and $Q_{n+1}$ use the sequence number *n*. Therefore, $R_{n}$ is the first response that appears after $Q_{n}$ and before $Q_{n+1}$. Let us assume $T_{proc}\ll RRP$ and $T_{t+r}\ll RRP$. Then, the defining characteristics of these two orders can be summarized as follows:

(1) Forward order
  (a) Has the event timeline: $Q_{n}≺V_{A}^{n}≺R_{n}≺V_{B}^{n}≺Q_{n+1}≺\cdots$, where ≺ denotes the “precedes" relation.
  (b) RTT<RRP can be achieved if we limit $T_{2}<RRP/2$. If $T_{2}$ is not controlled, RTT can exceed RRP, as Figure 1 exemplifies.
(2) Reverse order
  (a) Has the event timeline: $Q_{n}≺V_{A}^{n}\left\|V_{B}^{n}≺R_{n}≺Q_{n+1}≺V_{A}^{n+1}\right\|V_{B}^{n+1}≺R_{n+1}≺\cdots ⟹Q_{n}≺V_{B}^{n}≺R_{n}$. Here, x‖y means “either x≺y or y≺x”.
  (b) Vehicle A experiences RRP<RTT<2·RRP regardless of how $T_{2}$ is set.

The intuitive meaning of a reverse order is a resource topology that forces the response to a request to be transmitted *after* the next request. Formally, it is identified by the existence of the precedence relation $Q_{n}≺V_{B}^{n}≺R_{n}$ in the timeline.

In the forward order, if $T_{2}$ is large, we can have RTT>RRP as shown by Figure 1. Still, it can be mitigated using a small $T_{2}$. For example, with $T_{2}<RRP/2$, we have

$$RTT\approx t\left(V_{A}^{n},Q_{n}\right)+t\left(V_{B}^{n},R_{n}\right)<2\cdot T_{2}<RRP,$$

where t(x,y)=x−y is the time difference between two events *x* and *y*. This is a necessary condition for a response to be obtained before the next request is transmitted in one RRP. In contrast to the forward order, where the interaction delay can be controlled below RRP, the sub-RRP delay is not feasible in the reverse order using only $T_{2}$. Notice that we have $Q_{n}≺V_{B}^{n}≺R_{n}$ in the reverse order, whereas in the forward order, we have $Q_{n}≺R_{n}≺V_{B}^{n}$. Then, in the reverse order, we must experience RTT>RRP because $R_{n}$ cannot use $V_{B}^{n}$ but use $V_{B}^{n+1}$. Specifically,

(2) $$\begin{array}{ccc} RTT & > & t(V_{B}^{n+1},Q_{n})\\ & > & t\left(V_{B}^{n+1},V_{B}^{n}\right)\;\;\;\;∵Q_{n}≺V_{B}^{n}\\ & = & RRP \end{array}$$

regardless of $T_{2}$. On the other hand, we also have in the reverse order

(3) $$\begin{array}{ccc} RTT & < & t\left(V_{B}^{n+1},R_{n}\right)+t\left(V_{A}^{n},Q_{n}\right)\\ & < & t\left(V_{B}^{n+1},V_{B}^{n}\right)+t\left(V_{A}^{n},Q_{n}\right)\\ & = & RRP+t(V_{A}^{n},Q_{n})\\ & \leq & 2\cdot RRP \end{array}$$

because $t\left(V_{A}^{n},Q_{n}\right)\leq T_{2}\leq RRP$. Notice that the maximum delay does not exceed 2·RRP even in the reverse order. We will confirm this in our measurements in Section 4.2. Although bounded, the bound can be unacceptably large for real-time safety-critical communication between interacting vehicles. In particular, it is much larger than the minimum sidelink communication delay.

**Figure 2:**
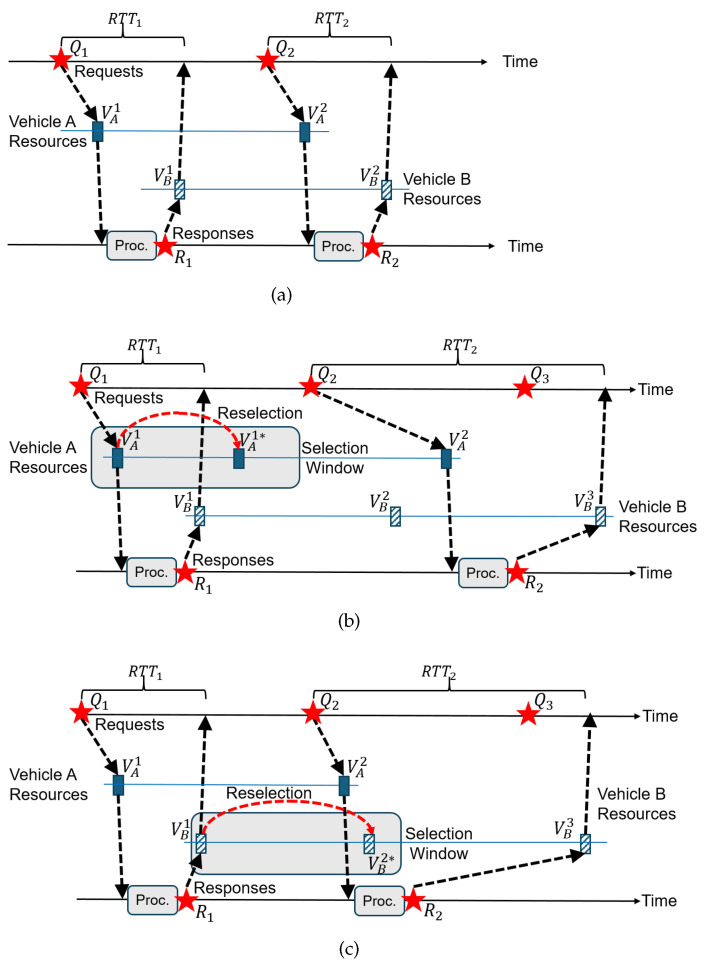
Resource topology: forward order *vs.* reverse order: (**a**) Forward order. (**b**) Reverse order caused by A’s reselection. (**c**) Reverse order caused by B’s reselection.

Figure 2 illustrates the two resource topologies with examples and shows how reverse orders are formed by SPS resource reselections under some unfortunate series of events. In Figure 2b, the reverse order is caused by the requesting vehicle. Upon using $V_{A}^{1}$, its RC value reached zero, so it performs a reselection. Inside the selection window, suppose the new resource $V_{A}^{1*}$ is situated closer to the right edge so that we have $V_{B}^{1}≺V_{A}^{1*}$. Here, “*” marks reselected resource. Since $V_{A}^{1*}≺Q_{2}$ due to the selection window size limit, $Q_{2}$ must use $V_{A}^{2}$ where $t(V_{A}^{2},V_{A}^{1*})=RRP$. Still, the resources at B have not changed their positions and maintain the same precedence relation with requests. In particular, $Q_{2}≺V_{B}^{2}$. Then, we have four events $V_{B}^{2}≺V_{A}^{2}≺R_{2}≺V_{B}^{3}$ that span an RRP. Therefore, $RTT>t(V_{B}^{3},Q_{2})>RRP$. In contrast, in Figure 2c, it is the replier that performs the reselection that causes a reverse order. Here, the replier’s resource shifted from extreme left to extreme right in the selection window. It could be $V_{B}^{2*}≺V_{A}^{2}$, but we considered it in Figure 2b, so we will assume that $V_{A}^{2}≺V_{B}^{2*}$ here. Due to the selection window size limit, we have $Q_{2}≺V_{B}^{2*}≺R_{2}$, i.e., a reverse order. Therefore, $R_{2}$ must use $V_{B}^{3}$ instead, where $t(V_{B}^{3},V_{B}^{2*})=RRP$. Since $Q_{2}≺V_{B}^{2*}$, $t(V_{B}^{3},Q_{2})>RRP$. We could also consider the case where the new resource is located prior to $Q_{2}$ so that we have $V_{B}^{1*}≺Q_{2}$. This case, however, is not a reverse order. Notice that the new resource is denoted by $V_{B}^{1*}$, not $V_{B}^{2*}$. Then, we have $R_{1}≺V_{B}^{1}≺V_{B}^{1*}≺Q_{2}≺\cdots$, which will hold for the next sequence, hence $Q_{2}≺V_{A}^{2}≺R_{2}≺V_{B}^{2}$, i.e., a forward order.

Unfortunately, the reverse order formation cannot be avoided. This is because, in SPS, probabilistic resource re-allocations are continually performed in order to mitigate resource collisions among vehicles. Whenever the internal RC count decreases to zero, the vehicle performs a reselection with a probability of $1-P_{keep}$. Even if the resource topology for a pair of interacting vehicles has been in a forward order, the reselection by one of the vehicles can cause a reverse order as Figure 2b,c demonstrate.

### 4.2. Validation Through Real-Life Measurements

**Figure 3:**
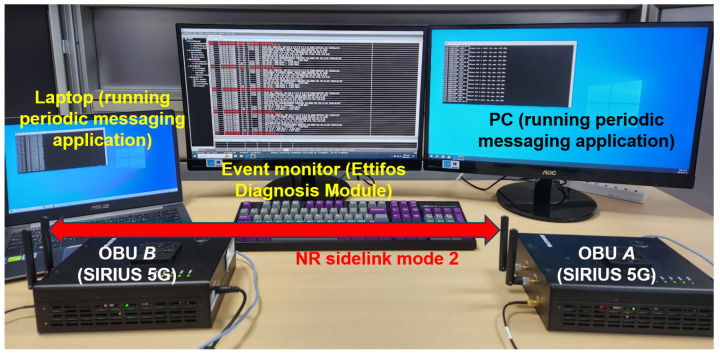
Configuration for V2V communication delay measurements using commercial cellular V2X OBU devices.

To verify the aspects of interaction delay discussed above, we conducted measurement experiments using commercial cellular V2X equipment. Specifically, we observed the impact of the large selection window size and the reverse order. The commercial V2X devices used in the request-response interaction are two units of Ettifos SIRIUS OBU [38], which are 3GPP Release-16 compliant 5G-V2X sidelink UE implemented in software-defined radio (SDR). Figure 3 shows the configuration used in the measurements.

SIRIUS OBUs *A* and *B* were, respectively, connected to laptop $A_{1}$ and desktop $B_{1}$ through Ethernet for application execution. For event monitoring, a proprietary software called Ettifos Diagnosis Module was used. Following the message generation by $A_{1}$, SIRIUS *A* periodically transmitted packets carrying the messages on the NR sidelink with inter-transmission time (ITT) values ranging from 100 ms to 500 ms at 100 ms intervals and a packet size of 300 bytes, while time-stamping on each packet for RTT monitoring. SIRIUS *B* received the packet from the sidelink and simply echoed the packet back on the sidelink. The focus of this paper is on the communication latency caused by resource scheduling and event precedence. We put the devices at a close distance in line-of-sight (LoS) during measurements.

Depending on the type of interactive applications, processing (“Proc.” in Figure 1 and Figure 2) could be more involved. However, in the current experiments, we let $B_{1}$ simply echo the request message, so $T_{proc}\approx 0$. Then, we measured RTT at $A_{1}$. We collected 1000 RTT samples between *A* and *B* for different ITT values. The parameters used in the sidelink communication are summarized in Table 1, which meet the specified values in the 3GPP Release 16.

**Figure 4:**
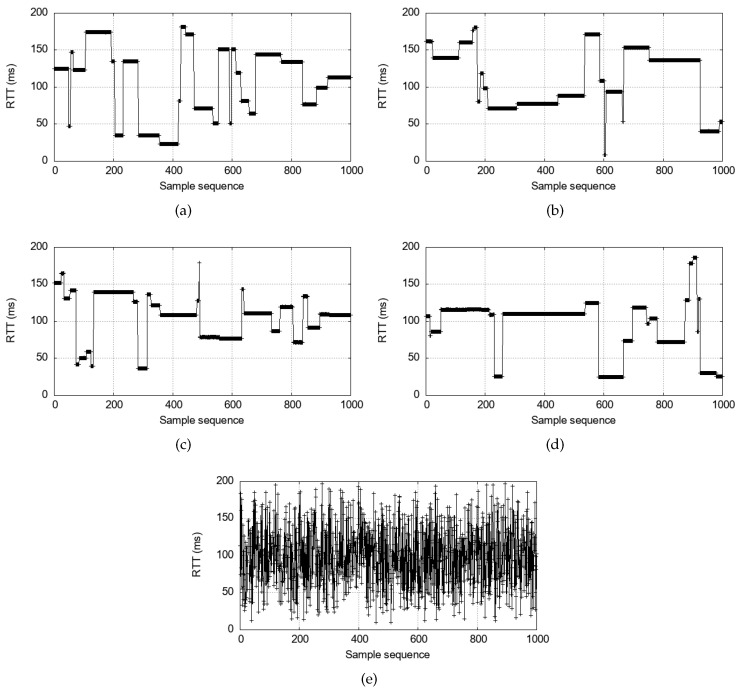
Real-life RTT values experienced between two SIRIUS devices communicating through periodic transmissions for 100 seconds, under various ITT values; $T_{2}=100$ ms, PDB=100 ms: (**a**) ITT = 100 ms. (**b**) ITT = 200 ms. (**c**) ITT = 300 ms. (**d**) ITT = 400 ms. (**e**) ITT = 500 ms.

The measured end-to-end RTTs using the commercial V2X device are presented in Figure 4. The minimum latency is observed at approximately 20 ms when the packets are sent at 10 Hz in Figure 4a. Interestingly, however, there are frequent delay spikes that exceed an RRP (i.e., 100 ms). Since $T_{2}$ is not controlled but set at 100 ms, these spikes can be caused in either forward order or reverse order. What is confirmed from this real-life measurement with RRP=100 ms and $T_{2}=100$ ms is that the round-trip delay dynamics are highly variable, unpredictable, and excessive for time-critical applications. This is because resource reselections are independently executed on the two communicating sides. Also, notice that the high delay spikes never exceed 2·RRP as shown in Equation (3).

Even if we lower the messaging rates, Figure 4b,d show that the variable delay spikes problem does not disappear. This is because the delay is caused by the scheduling delay, not the queueing delay from the high traffic load. An interesting qualitative change happens when the messaging rate is 2 Hz, in Figure 4e. Here, reselections are extremely frequent, and the high delay plateaus do not appear. This is because of the ReselectAfter setting. If a vehicle fails to use the number of ReselectAfter=4 reserved resources consecutively, it must reselect a new resource for the next transmission [7]. Therefore, when ITT=500 ms in Figure 4, every transmission triggers a reselection. It means that the reverse order never happens because $Q_{n}≺R_{n}≺V_{B}^{n}$ always holds because of the triggered reselection. However, the reselection upon every transmission is highly undesirable because it disrupts the SPS algorithm and can cause lower packet reception ratios [39]. Moreover, large ITT values are generally not suitable for real-time interactions between vehicles such as platooning [19].

Finally, we note that many delay spikes appear as wide plateaus. It implies that the resource topology between *A* and *B* is maintained for an extended period of time once formed. For example, the delay spikes caused by the reverse order can last until a resource reselection takes place on either device, changing the reverse order to a forward order. Consequently, the reverse order could span a few seconds. In general, for the given resource keep probability $P_{keep}=1-p$, the number of times *l* that a vehicle keeps the same resource is geometrically distributed. Suppose $l_{A}$ and $l_{B}$ are the numbers of such consecutive resource uses by the two vehicles that happened to interact. When such an encounter is made, the remaining resource-keeping times are independent of how long the vehicles have been using the frequency resource because the run length is a geometric random variable (i.e., memoryless). Then, regardless of when the encounter occurs, the mean of the shorter of the two remaining reuse times, $\min(l_{A},l_{B})$, is

$$E\left[\min\left(l_{A},l_{B}\right)\right]=\frac{1}{2p-p^{2}}.$$

At $P_{keep}=0.8$ for example, $E\left[\min\left(l_{A},l_{B}\right)\right]=2.\dot{7}$. With RRP≥100 ms and RC∼Uniform(5,15) [7], every time a resource is kept, it is used for one second on average. Therefore, the reverse order will persist for more than two seconds on average. Obviously, it could be worse. For example, the 90th percentile is 6 seconds. Moreover, even if one vehicle reselects, it can still result in another reverse order. In that case, the duration of the reverse order will persist even longer. Essentially, it is what Figure 4 demonstrated. Such long latency spikes can severely harm time-critical applications. Even more concerning, the unpredictable and significant delay fluctuations would pose a major challenge for real-time applications to absorb. Hence, we must find a solution to this problem.

## 5. Solution Approach

In this section, we develop a solution to solve the large, variable, and unpredictable delay problem in interactive communication between vehicles using periodic resources on the NR sidelink. Specifically, our solution comprises two components based on the discussions in the previous section. The first is reducing $T_{2}$ to address the large delay issue in the forward resource order. Recollect that it does not solve the problem for the reverse order. Therefore, the second component is detecting the reverse order and correcting it. The first is straightforward, so that we will focus on the second. In particular, we will attempt to develop a provably reliable method to solve the problem.

The root cause of the reverse order problem is the independent resource allocation on the two endpoints of a sidelink, whereas the application requires a bi-directional exchange of packets that implements request-response style interaction. Although the two peers could coordinate their resource allocations explicitly to prevent the reverse order, it will require an additional signaling protocol. Therefore, we consider here a standard-compliant scheme where the MAC layer at the replier side automatically eliminates the possibility of the reverse order without additional scheduling. The proposed solution employs the replier-side packet delay budget (PDB) and other parameters to promptly initiate resource re-alignment whenever there is a risk of reverse order forming.

### 5.1. Reselection Trigger at Replier to Prevent Reverse Orders

We consider the proposed replier behavior when the resource topology changes due to the SPS algorithm—first, when the requesting vehicle performs a resource reselection according to SPS, and second, when the replier itself performs the reselection according to SPS.

#### 5.1.1. Coping with the Reverse Order Caused by Reselection by A

**Figure 5:**
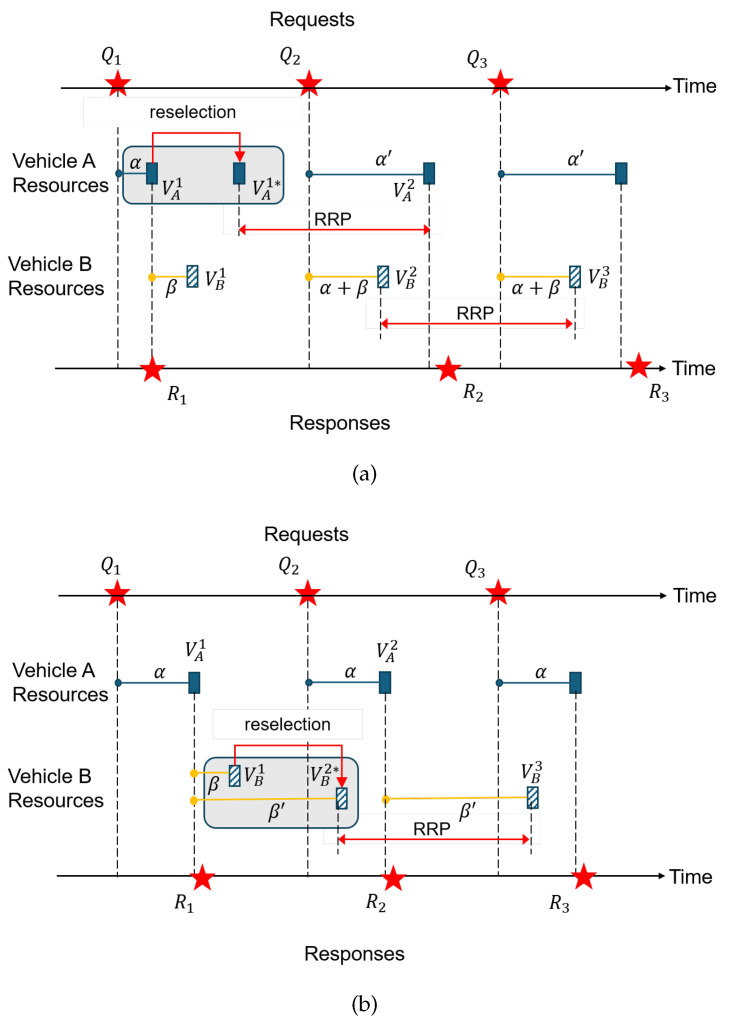
Reverse order caused by either communicating party: (**a**) Reselection by A. (**b**) Reselection by B.

In Figure 5a, Vehicle A and B start their interaction in a forward-order resource topology. Again, we set the time reference to requests. Suppose that Vehicle A must use $V_{A}^{2}$ for $Q_{2}$ as a result of the reselection performed after transmitting $Q_{1}$. Consequently, the resource order between two vehicles is changed to a reverse order: $Q_{2}≺V_{B}^{2}≺R_{2}$ in the sequence of $Q_{2}≺V_{B}^{2}≺V_{A}^{2}≺R_{2}≺Q_{3}≺V_{B}^{3}≺\cdots$. Since $t(V_{B}^{3},V_{B}^{2})=RRP$, we have $RTT>t(V_{B}^{3},Q_{2})>RRP$. In order to precisely describe RTT in terms of the system parameters in SPS, we define timing-related variables as follows: $\alpha =t(V_{A}^{1},Q_{1})$, $\beta =t(V_{B}^{1},R_{1})$. Notice that the relations between variables in Figure 5a before reselection is given as

(4) $$\begin{array}{ccc} T_{1(A)}\leq & \alpha & \leq T_{2(A)},\\ T_{1(B)}\leq & \beta & \leq T_{2(B)}\\ ⟹T_{1(A)}+T_{1(B)}\leq & \alpha +\beta & \leq T_{2(A)}+T_{2(B)}. \end{array}$$

After the reselection that caused the reverse order, we additionally have

(5) $$\alpha +\beta <\alpha^{\prime }\leq T_{2(A)}.$$

Let δ denote the time for Vehicle B’s response message $R_{2}$ to wait until the next available resource. Then, from Figure 5a,

$$\delta =\left(\alpha +\beta +RRP\right)-\alpha^{\prime }-T_{proc}$$

where $T_{proc}$ is the request processing delay at the replier. From (4) and (5), we know the minimum value of δ as

(6) $$\begin{array}{ccc} \delta & \geq & RRP+T_{1(A)}+T_{1(B)}-\alpha^{\prime }-T_{proc}\\ & \geq & RRP+T_{1(A)}+T_{1(B)}-T_{2(A)}-T_{proc} \end{array}$$

which is described only in terms of the system parameters except $T_{proc}$, irrespective of α, β, $\alpha^{\prime }$, and δ. Or, if we have the same $T_{1}$ and $T_{2}$ values on both vehicles for their common application, we have the minimum value of δ as:

(7) $$\delta \geq RRP+2T_{1}-T_{2}-T_{proc}$$

when the reverse order is caused by the requesting vehicle’s resource reselection. For convenience, we will call the RHS by $\delta_{min}$. Because $T_{proc}$ depends on the processing capacity, not the communication protocol, it is a local parameter at the replier vehicle *B*. Since it is expected to be minimal, we set $T_{proc}\approx 0$ in this paper. If not, the replier can take account of $T_{proc}$ in (7). Notice that even if we reduce $T_{2}$ to a small value, $\delta_{min}$ only grows, which is one reason that small $T_{2}$ cannot be a solution to the reverse order.

The solution to break the condition in (7) that we propose in this paper is to trigger a reselection so that the reverse order is corrected. For this purpose, we exploit PDB, a parameter to the resource allocation layer. Specifically, we trigger a reselection when the condition (7) takes place by requiring

(8) $$\begin{array}{ccc} PDB & < & \delta_{min}\\ & = & RRP+2T_{1}-T_{2}-T_{proc}. \end{array}$$

At the replier Vehicle B, a reselection according to Equation (8) will always succeed to eliminate delay spikes. In the standard, the PDB-triggered reselection is a legitimate but optional feature: “*If the remaining PDB is not met, it is left for UE implementation whether to perform … sidelink resource reselection*” (3GPP TS 38.321 [7], 5.22.1.2). Below, we use Equation (8) in our experiments to validate that this approach works in a commercial V2X device.

What is important is that Equation (8) is free of the timing-related conditions represented by $\alpha ,\beta ,\alpha^{\prime }$. Consequently, the replier vehicle does not need to communicate with the requesting vehicle to query the values of α and $\alpha^{\prime }$ that are internal to the requestor. By simply using the parameters $T_{1}$ and $T_{2}$ specific to the application instead, the replier can correct the reverse order to a forward order.

Notice that as $T_{2}$ is set small in our proposal, the required change in PDB also becomes small, minimally affecting the originally specified PDB value. However, below, we will also discuss the impact of using larger $T_{2}$ values. As to reducing the PDB requirement on the MAC layer, we can consider its impact on the system in two ways. First, PDB is the application feature and is given to the MAC layer as a parameter. Because the PDB requirement is the worst-case delay bound, meeting it earlier does not pose a problem, unlike violating it in the opposite direction. The second impact is the increase of reselections. As we more drastically reduce PDB in Equation (8), the reselection may become more rampant. Because the increased reselections can affect the packet reception ratio (PRR) in the SPS-based system [39], it is important to minimize the PDB requirement reduction. However, we will show in the experiments below that the number of reselections does not significantly increase under our proposal. Recollect that it was the duration of the delay spikes that is more concerning than their frequency (see Figure 4).

#### 5.1.2. Coping with the Reverse Order Caused by Reselection by B

Now, let us consider the second type of reverse order, which is created by the replier’s reselection (Figure 5b). In this case, the replier does not have to indirectly infer the reverse order condition. This is because the replier knows immediately whether or not a selected resource would create a reverse order. In Figure 5b, for instance, B knows that $Q_{2}$ will arrive at one RRP after $V_{A}^{1}$. Therefore, when B performs a reselection after transmitting $R_{1}$, it would reject $V_{B}^{2*}$ as depicted in the figure because it will create a reverse order: $Q_{2}≺V_{B}^{2*}≺V_{A}^{2}≺R_{2}≺\cdots$. Instead, B will attempt to use a resource situated more towards the left edge of the selection window. Unfortunately, B does not know the value of α that is internal to A, nor the timing of $Q_{2}$. Therefore, the best strategy would be to use a small $T_{2}$ value so that the selected resource comes closer to the left edge of the selection window. Please note that because $T_{1}\leq \alpha ,\beta ,\beta^{\prime }\leq T_{2}$, a small $T_{2}$ will also reduce α, which will further help to make $V_{B}^{1*}≺Q_{2}$ happen. If we can ignore other delay components $T_{proc}$ and $T_{t+r}$, $T_{2}<50$ ms will guarantee $V_{B}^{1*}≺Q_{2}$; otherwise, $T_{2}$ should be accordingly reduced. Then, we will have $Q_{2}≺V_{A}^{2}≺R_{2}≺V_{B}^{2}≺Q_{3}$, where $t(V_{B}^{2},V_{B}^{1*})=RRP$. This is another reason that a small $T_{2}$ value should be used in our proposed solution. Thus, we have achieved $Q_{2}≺R_{2}≺V_{B}^{2}≺Q_{3}$, which is a forward order and satisfies RTT<RRP.

Please note that the resource re-alignment occurs for each vehicle pair. Usually, the maneuver coordination will be between two vehicles, so the complexity of re-alignment is not high. Even in the case of a 1-to-*N* relation in a single maneuver coordination session (e.g., leader-members communication in platooning), the *N* pairs can be separately performed as they are orthogonal to each other.

### 5.2. Evaluation

In this section, we evaluate the performance of the proposed scheme. We first execute our solution on the standard-compliant commercial devices and check if the proposed solution indeed removes the delay spikes. Then, we conduct additional simulations because the commercial product does not allow us maximum freedom for parameter configuration.

#### 5.2.1. Measurements

We test the proposed scheme on the same commercial OBUs from the measurement study in Section 4 with most of the configuration maintained as in Table 1. In order to apply Equation (8), however, we change $T_{2}$ and PDB here. Unfortunately, the SIRIUS device does not yet allow us full flexibility to adjust these two parameters; instead, it provides a few “application profiles” that set the two parameters are selected values. In particular, it allows two values for $T_{2}$: 10 ms and 100 ms; for PDB, four values: 80 ms, 90 ms, 95 ms, and 100 ms. We will test with these values in the measurement experiments, and then for other values of PDB, perform a simulation with the configuration in Table 1.

**Figure 6:**
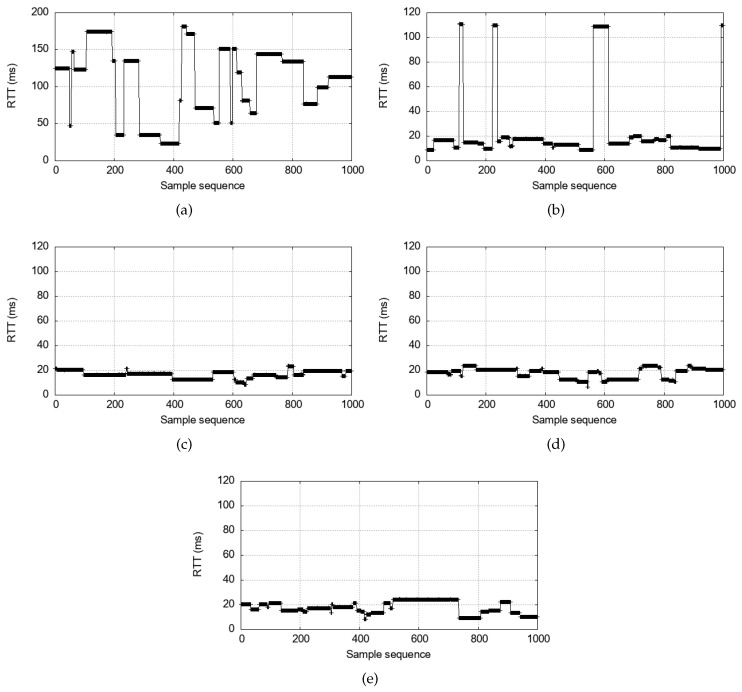
Comparison of bi-directional communication latency measured on commercial V2V device pair: (**a**) Baseline: $T_{2}=100$ ms, PDB=100 ms. (**b**) $T_{2}=10$ ms, PDB=100 ms. (**c**) $T_{2}=10$ ms, PDB=95 ms. (**d**) $T_{2}=10$ ms, PDB=90 ms. (**e**) $T_{2}=10$ ms, PDB=80 ms.

Figure 6 presents the measurement result for V2V interactive communication under the current SPS algorithm as baseline *vs.* under the proposed scheme. Compared to the baseline ($T_{2}=100$ ms and PDB=100 ms) that produces frequent and unpredictable delay spikes as in Figure 6a, reducing $T_{2}$ (Figure 6b) has two noticeable effects. First, it reduces the base delay to approximately 20 ms, which is expected. With $T_{proc}$ and $T_{t+r}$ relatively much smaller, we have $RTT\approx 2\cdot T_{2}$ in Equation (1). Second, the delay spikes are significantly reduced in number. However, it alone does not eliminate the spikes. This is because small $T_{2}$ alone cannot resolve the reverse order. Indeed, the remaining delay spikes are the ones caused by the reverse order. In contrast, the PDB-triggered reselection as guaranteed by Equation (8) combined with the small $T_{2}$ eliminates the delay spikes as shown in Figure 6d,e. For instance, PDB=80 ms satisfy the requirement in Equation (8) for the PDB-triggered reselection, and the reverse order instances are successfully removed.

It is remarkable that Figure 6c shows that even a larger PDB value than the conservative requirement of (8) may still eliminate the delay spikes. Equation (8) informs us that under the given parameter values ($RRP,\;T_{1},\;T_{2}$), any PDB value of less than $RRP+2T_{1}-T_{2}=92$ ms will trigger a PDB-based reselection to eliminate delay spikes. However, it is a conservative bound that will guarantee a reselection. Specifically, in some cases, a PDB-triggered reselection can still take place for larger PDB values than the bound. This is because Equation (8) is assuming the worst case $\alpha^{\prime }=T_{2}$, whereas $\alpha^{\prime }<T_{2}$ is allowed in Equation (6) as long as $\alpha^{\prime }>\alpha +\beta$ as given in Equation (5). For example, if RRP=100,$T_{1}=1,T_{2}=10$, the PDB bound is 92 ms, but PDB=95 ms will still trigger a reselection for $\alpha =1,\beta =1,\alpha^{\prime }=3$. This is because the time difference from the resource α+β to the next resource is $RRP+\left(\alpha +\beta \right)-\alpha^{\prime }=99$ ms, which exceeds 95 ms of non-conservative PDB value. Finally, Figure 6 suggests that once Equation (8) is satisfied, PDB does not have to be unnecessarily lowered as all PDB values 90 ms and below produce comparable delay performance. It will be corroborated by the simulation study below that attempts to use other PDB values. The simulation will also show that excessively low PDB values may only lead to more reselections, although the increase is marginal.

Table 2 summarizes the measured delay statistics. It is evident that reducing the PDB below the PDB of 100 ms significantly lowers the delay standard deviation, indicating the elimination of delay spikes. In contrast, reducing only $T_{2}=10$ ms with the default PDB slightly decreases the mean delay, but the reduction in deviation is not as pronounced because not all delay spikes are eliminated.

#### 5.2.2. Supplementary Simulation Experiments

**Figure 7:**
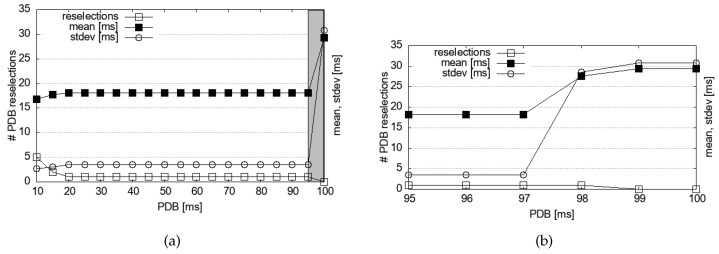
Detailed PDB simulation results regarding delay and reselection statistics; $T_{2}=10$ ms: (**a**) Overall dynamics, 10≤PDB≤100. (**b**) Detailed dynamics at 95≤PDB≤100.

**Figure 8:**
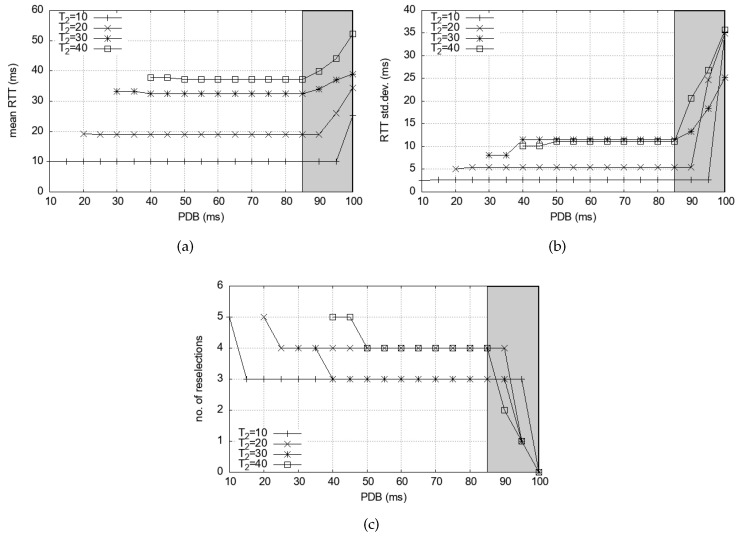
Impact of $T_{2}$ values on mean delay and delay variance in simulation: (**a**) Mean RTT. (**b**) RTT standard deviation. (**c**) PDB-triggered reselections per 1000 packet transmissions.

To further confirm that Equation (8) completely removes the delay spikes in other PDB values, we need to test various PDB values. Also, we want to explore the impacts of other $T_{2}$ values than 10 ms and 100 ms. However, the SIRIUS OBU we used does not allow free variations of these parameters, so we resorted to simulation. Additionally, simulations allow us to gather statistics on PDB-triggered reselections that are not revealed by the commercial device. Using an in-house simulator with the same configuration as in Table 1, we collected 1000 RTT samples, which are presented in Figure 7 and Figure 8.

Figure 7a confirms that the delay dynamics begin to improve almost immediately as PDB decreases from 100 ms. Figure 7b provides detailed data. A PDB value as high as 97 ms can achieve comparable mean delay and deviation to the more conservatively set PDB value in Equation (8).

Excessive reselections can undermine the predictability of resource use in the SPS algorithm, leading to increased packet collisions and adversely affecting the packet reception ratio (PRR) [39], which in turn may impact application performance. Therefore, PDB-based reselections must be kept minimal to reduce their impact on the PRR of other vehicles and applications. Figure 7 shows that reselection increases are indeed minimal, and the system remains stable under the proposed scheme until the PDB value is extremely low. Therefore, we argue that under our scheme reselections are not excessive, so it would minimally affect the PRR of other vehicles.

Finally, we investigate the impact of $T_{2}$ value in Equation (8). As to how small $T_{2}$ can be, the standards specify $T_{2min}\leq T_{2}$. For LTE V2X, 10 ms is a mandatory minimum value for $T_{2}$ in 3GPP Release 15. In Release 16, $T_{2min}\left(prio_{TX}\right)\leq T_{2}\leq 100$, where $T_{2min}$ is a function of the priority of the packet to transmit [40], which is provided by the higher layers. For NR V2X, more low-latency configuration has become possible. In 3GPP Release 16, $T_{2min}$ can be set to 1 ms, 5 ms, 10 ms, or 20 ms (i.e., *sl-SelectionWindowList-r16*) with 15 kHz subcarrier spacing [41]. Therefore, those interactive applications that require low latency could use these small $T_{2}$ values. However, if larger $T_{2}$ values are desirable for any reason, Equation (8) tells us that PDB needs to be accordingly reduced. It will affect the mean delay, delay variance, and the number of reselections. Therefore, we investigate the impact of larger $T_{2}$ values on these performance measures. A $T_{2}$ value that is too large contradicts the purpose of having an interaction delay smaller than an RRP, so we only consider $T_{2}<50$ ms in this paper. Also, note that we restricted $PDB\geq T_{2}$ because PDB-triggered reselections will be automatically performed if the selected resource is situated in slot *n*, $PDB<n\leq T_{2}$. Figure 8 presents the mean RTT and the deviation.

We notice that as $T_{2}$ increases, the mean delay rises accordingly in Figure 8a. Even for larger $T_{2}$ values, the mean delay stabilizes as the PDB is set to 85 ms or smaller. For these $T_{2}$ values, the PDB values are larger than the conservative bound of Equation (8). In Figure 8b, the high variability in PDB values over 85 ms signals the existence of delay spikes. Reducing PDB triggers reselections by Equation (8), and removes the delay spikes. These increasing number of reselections in Figure 8c are the cost for the removal, but their numbers are relatively small. This is because the delay spikes last long once they occur, but they are not frequent.

In summary, to address the issues of highly variable RTTs and delay spikes in interactive vehicular communication, we suggest that two measures be taken in combination. First, the selection window size should be set according to $T_{2}<RRP/2$. This configuration maximizes the likelihood that a requesting vehicle will receive a response before its next request transmission by limiting the selection window size in Equation (1). Second, to eliminate the remaining delay spikes still not removed by the first measure, PDB-triggered reselection should be enforced by setting $PDB<\delta_{min}$ in Equation (8).

### 5.3. Interactions and Dependencies

The proposed solution operates within the standard resource allocation algorithm, namely the Sensing-Based Semi-Persistent Scheduling (SB-SPS) [6], and it only affects the implementation-dependent parts. The SB-SPS is used for distributed resource allocation for the sidelink Mode 2, which is the base mode, as the infrastructure, such as the base station, may be unavailable on the roadside. The SB-SPS algorithm for the sidelink Mode 2 runs only on onboard units (OBUs). As the parameters that our proposed scheme uses come from the application through the SB-SPS algorithm, there is no new interface to be defined, but it can access the parameters like RRP, PDB, $T_{1}$, and $T_{2}$ from the SB-SPS algorithm itself. Therefore, the only dependency that the proposed scheme has is on the SB-SPS algorithm to access those parameters.

## 6. Conclusions

This paper shows through real-life V2X device experimentation, analysis, and simulation that under periodic message transmissions such as in maneuver coordination or platoon coordination, the interacting vehicles can frequently and unpredictably experience variable delay and large delay spikes that can last for a few seconds at a time. This is a fundamental issue as long as resource allocations and re-allocations are performed independently on interacting vehicles. The problem is not solved by simply tightening the selection window (i.e., small $T_{2}$ value) in the Semi-Persistent Scheduling (SPS) algorithm. We develop a standard-compliant solution approach that exploits packet delay budget (PDB)-triggered reselection coupled with a small selection window size and derive a concrete condition under which the reselection should be triggered. The small selection window in the SPS algorithm has an effect only when this condition is correctly applied. We demonstrate through measurement and simulation that the proposed scheme effectively eliminates the delay spikes, keeping the interaction latency low. Also, the required PDB-triggered reselections are minimal, with a low potential to disrupt the SPS operation at other vehicles. We believe that the significance of this work is to show that low interaction delay is possible under standard periodic scheduling that has been adopted as the base mode through generations of use cases in vehicular communication.

## Figures and Tables

**Table 1:** 5G-V2X sidelink parameters configuration for measurements on SIRIUS devices (for Figure 4).

Parameter	Value
Transmit power	20 dBm
Subcarrier spacing	15 kHz
Channel bandwidth	20 MHz
Carrier frequency	5865 MHz
Subchannel size	15
*NumSubchannel*	7
Bandwidth	20 MHz
PSCCH time resources	2
PSCCH frequency resources	10
MCS index	8
*ReselectAfter*	4
$T_{2}$	100 ms
Packet size	300 B
Packet delay budget (PDB)	100 ms
Resource Reservation Period (RRP)	100 ms
Resource keep probability ($P_{keep}$)	0.8
Inter-Transmission Time (ITT)	[100, 200, 300, 400, 500] ms

**Table 2:** Measured delay mean (μ) and deviation (σ) for configurable $T_{2}$ and PDB values on SIRUIS device pair.

Scheme	μ [ms]	σ [ms]
Baseline ($T_{2}=100$ ms, PDB=100 ms)	102.48	45.61
$T_{2}=10$ ms, PDB=100 ms	27.27	33.14
$T_{2}=10$ ms, PDB=95 ms	15.70	3.78
$T_{2}=10$ ms, PDB=90 ms	16.87	3.96
$T_{2}=10$ ms, PDB=80 ms	16.26	4.23

## Data Availability

The original contributions presented in the study are included in the article, further inquiries can be directed to the corresponding author.

## References

[1] Hussein N.H., Yaw C.T., Koh S.P., Tiong S.K., Chong K.H. (2022). A Comprehensive Survey on Vehicular Networking: Communications, Applications, Challenges, and Upcoming Research Directions. IEEE Access.

[2] Filho J.G., Patel A., Batista B.L.A., Celestino J. (2016). A systematic technical survey of DTN and VDTN routing protocols. Comput. Stand. Interfaces.

[3] Dias J.A.F.F., Rodrigues J.J.P.C., Kumar N., Saleem K. (2015). Cooperation strategies for vehicular delay-tolerant networks. IEEE Commun. Mag..

[4] (2012). IEEE Standard for Information Technology–Telecommunications and Information Exchange Between Systems Local and Metropolitan Area Networks—Specific Requirements Part 11: Wireless LAN Medium Access Control (MAC) and Physical Layer (PHY) Specifications.

[5] (2016). IEEE Standard for Wireless Access in Vehicular Environments (WAVE)—Networking Services.

[6] 3GPP Technical Specification Group Radio Access Network; NR; Medium Access Control (MAC) Protocol Specification (Release 18), TS 38.214. https://portal.3gpp.org/desktopmodules/Specifications/SpecificationDetails.aspx?specificationId=3216.

[7] 3GPP Technical Specification Group Radio Access Network; NR; Medium Access Control (MAC) Protocol Specification (Release 16), TS 38.321. https://portal.3gpp.org/desktopmodules/Specifications/SpecificationDetails.aspx?specificationId=3194.

[8] Euro NCAP 2025 Roadmap: IN PURSUIT OF VISION ZERO. https://cdn.euroncap.com/media/30700/euroncap-roadmap-2025-v4.pdf.

[9] Chen X., Deng Y., Ding H., Qu G., Zhang H., Li P., Fang Y. (2024). Vehicle as a Service (VaaS): Leverage Vehicles to Build Service Networks and Capabilities for Smart Cities. IEEE Commun. Surv. Tutorials.

[10] US NTSB Collision Between a Car Operating with Automated Vehicle Control Systems and a Tractor-Semitrailer Truck Near Williston, Florida, May 7, 2016.

[11] Clarke L. How Self-Driving Cars Got Stuck in the Slow Lane. https://www.theguardian.com/technology/2022/mar/27/how-self-driving-cars-got-stuck-in-the-slow-lane.

[12] Hajbabaie A., Tajalli M., Bardaka E. (2024). Effects of Connectivity and Automation on Saturation Headway and Capacity at Signalized Intersections. Transp. Res. Rec..

[13] Lekidis A., Bouali F. C-V2X network slicing framework for 5G-enabled vehicle platooning applications. Proceedings of the IEEE Vehicular Technology Conference (VTC2021-Spring).

[14] Kianfar R., Falcone P., Fredriksson J. (2015). A control matching model predictive control approach to string stable vehicle platooning. Control. Eng. Pract..

[15] Kolat M., Bécsi T. (2008). Multi-Agent Reinforcement Learning for Highway Platooning. Electronics.

[16] ETSI Intelligent Transport Systems (ITS); Vehicular Communications; Basic Set of Applications; Part 2: Specification of Cooperative Awareness Basic Service, TS 302 637-2 V1.4.1. https://www.etsi.org/deliver/etsi_en/302600_302699/30263702/01.04.01_30/en_30263702v010401v.pdf.

[17] ETSI Intelligent Transport Systems (ITS); Vehicular Communications; Basic Set of Applications; Collective Perception Services, TS 103 324 V2.1.1. https://www.etsi.org/deliver/etsi_ts/103300_103399/103324/02.01.01_60/ts_103324v020101p.pdf.

[18] ETSI Intelligent Transport Systems (ITS); Vehicular Communication; Basic Set of Application; Maneuver Coordination Service, TS 103 561 V0.0.1. https://portal.etsi.org/webapp/WorkProgram/Report_WorkItem.asp?WKI_ID=53496.

[19] ETSI Intelligent Transport Systems (ITS); Platooning; Informative Report for Platooning. Technical Report TR 103 439 V2.1.1. https://www.etsi.org/deliver/etsi_tr/103400_103499/103439/02.01.01_60/tr_103439v020101p.pdf.

[20] ENSEMBLE Consortium D3.1 Detailed Design of the Unbranded Tactical Layer. https://platooningensemble.eu/storage/uploads/documents/2023/03/13/ENSEMBLE-D3.1-Detailed-design-of-the-unbranded-Tactical-Layer_FINAL.pdf.

[21] Gray J.N., Bayer R., Graham R.M., Seegmüller G. (1978). Notes on data base operating systems. Operating Systems. Lecture Notes in Computer Science.

[22] ETSI Intelligent Transport Systems (ITS); Vehicular Communications; Basic Set of Applications; Maneuver Coordination Service; Release 2, TS 103 561 V0.0.2. https://portal.etsi.org/webapp/WorkProgram/Report_WorkItem.asp?WKI_ID=53496.

[23] Tripp-Barba C., Zaldívar-Colado A., Urquiza-Aguiar L., Aguilar-Calderón J.A. (2019). Survey on Routing Protocols for Vehicular Ad Hoc Networks Based on Multimetrics. Electronics.

[24] Mezher A.M., Igartua M.A. (2017). Multimedia Multimetric Map-Aware Routing Protocol to Send Video-Reporting Messages over VANETs in Smart Cities. IEEE Trans. Veh. Technol..

[25] Al-Shareeda M.A., Anbar M., Hasbullah I.H., Manickam S. (2020). Survey of authentication and privacy schemes in vehicular ad hoc networks. IEEE Sensors J..

[26] Belamri F., Boulfekhar S., Aissani D. (2020). A survey on QoS routing protocols in Vehicular Ad Hoc Network (VANET). Telecommun. Syst..

[27] Cooper C., Franklin D., Ros M., Safaei F., Abolhasan M. (2016). A Comparative Survey of VANET Clustering Techniques. IEEE Commun. Surv. Tutorials.

[28] Wu Q., Wang W., Fan P., Fan Q., Wang J., Letaief K.B. (2024). URLLC-Awared Resource Allocation for Heterogeneous Vehicular Edge Computing. IEEE Trans. Veh. Technol..

[29] ETSI Intelligent Transport Systems (ITS); Platooning; Pre- Standardization Study. Technical Report TR 103 298 V0.0.5. https://portal.etsi.org/webapp/workProgram/Report_WorkItem.asp?wki_id=44191.

[30] He Y., Wu B., Dong Z., Wan J., Shi W. (2023). Towards C-V2X Enabled Collaborative Autonomous Driving. IEEE Trans. Veh. Technol..

[31] SAE International V2X Communications Message Set Dictionary, J2735. https://www.sae.org/standards/content/j2735_202007/.

[32] ETSI Intelligent Transport Systems (ITS); Vehicular Communications; Maneuvre Coordination Service (MCS); Pre-Standardization Study; Release 2. Technical Report TR 103 578 V2.1.1. https://www.etsi.org/deliver/etsi_TR/103500_103599/103578/02.01.01_60/tr_103578v020101p.pdf.

[33] Correa A., Avedisov S.S., Sepulcre M., Sakr A.H., Molina-Masegosa R., Altintas O., Gozalvez J. On the Impact of V2X-based Maneuver Coordination on the Traffic. Proceedings of the IEEE Vehicular Technology Conference (VTC2021-Spring).

[34] SAE International Application Protocol and Requirements for Maneuver Sharing and Coordinating Service, J3186. https://www.sae.org/standards/content/j3186_202303/.

[35] Molina-Masegosa R., Gozalvez J. (2017). LTE-V for Sidelink 5G V2X Vehicular Communications: A New 5G Technology for Short-Range Vehicle-to-Everything Communications. IEEE Veh. Technol. Mag..

[36] Nardini G., Virdis A., Campolo C., Molinaro A., Stea G. (2018). Cellular-V2X Communications for Platooning: Design and Evaluation. Sensors.

[37] Hegde S., Blume O., Shrivastava R., Bakker H. Enhanced resource scheduling for platooning in 5G V2X systems. Proceedings of the IEEE 5G World Forum (5GWF).

[38] Ettifos World’s First 3GPP Release 16-Compliant 5G-V2X Sidelink Platform SIRIUS. https://www.ettifos.com/product-sirius.

[39] Yin J., Hwang S.-H. (2022). Adaptive sensing-based semiper- sistent scheduling with channel-state-information-aided reselection prob- ability for LTE-V2V. ICT Express.

[40] 3GPP Technical Specification Group Radio Access Network; Evolved Universal Terrestrial Radio Access (E-UTRA); Physical Layer Procedures (Release 16), TS 36.213. https://portal.3gpp.org/desktopmodules/Specifications/SpecificationDetails.aspx?specificationId=2427.

[41] 3GPP Technical Specification Group Radio Access Network; NR; Radio Resource Control (RRC); Protocol Specification (Release 16), TS 38.331. https://portal.3gpp.org/desktopmodules/Specifications/SpecificationDetails.aspx?specificationId=3197.

